# Cucurbitacin D Induces Apoptotic Cell Death via NOX4 and Overcomes Radioresistance in Colorectal Cancer

**DOI:** 10.3390/ijms262412022

**Published:** 2025-12-13

**Authors:** Tae Woo Kim

**Affiliations:** Department of Biopharmaceutical Engineering, Dongguk University-WISE, 123 Dongdae-ro, Gyeongju 38066, Gyeongbuk, Republic of Korea; tae1410@naver.com or tae1410@dongguk.ac.kr; Tel.: +82-54-770-2224; Fax: +82-54-770-2519

**Keywords:** Cucurbitacin D, NOX4, apoptosis, ER stress, radiation

## Abstract

Colorectal cancer (CRC) incidence is a significant cancer globally, and radiotherapy resistance is a serious problem. Cucurbitacin D (CBD), extracted from many plants such as the tubers of *Trichosanthes kirilowii* and the fruits of *Ecballium elaterium* (squirting cucumber), has various therapeutic effects, such as anti-cancer, -inflammation, -diabetes, and -viral infection effects. Since reports have indicated that CBD exhibits effective anti-cancer activity across various cancer types, our hypothesis is that CBD will overcome radioresistance in CRC radiotherapy. In the present study, we identified that CBD, a triterpenoid compound isolated from *Trichosanthes kirilowii* and *Ecballium elaterium*, has an anti-cancer and anti-inflammatory effect in vivo and in vitro. In LPS-induced murine models, CBD suppresses LPS-mediated cytokines, including TNFα, IL-6, IL-1β, and COX-2. In CRC xenograft mouse models, CBD treatment results in significantly smaller tumor volumes than the control. In HCT116 and HT29 cells, CBD treatment suppresses cell viability and increases LDH cytotoxicity and caspase-3 activity and cleavage. However, combined treatment of CBD and Z-VAD-FMK inhibits caspase-dependent apoptosis and cell death. Since CBD induces intracellular calcium (Ca^2+^) and reactive oxygen species (ROS) generation, it mediates ER stress-induced apoptotic cell death through the PERK-ATF4-CHOP axis. Moreover, ER stress inducer thapsigargin (TG) mediates synergistic apoptotic cell death in CBD-treated HCT116 and HT29 cells. However, PERK or CHOP knockdown suppresses ER stress-mediated apoptosis in CBD-treated HCT116 and HT29 cells. CBD treatment induces oxidative stress through the NADPH Oxidase 4 (NOX4) and also increases ROS generation. However, NOX4 knockdown and ROS inhibitor NAC or DPI block ER stress-induced apoptotic cell death by inhibiting the suppression of cell viability and the elevation of caspase-3 activity, LDH cytotoxicity, and intracellular ROS activity in CBD-mediated HCT116 and HT29 cells. We established radioresistant CRC models (HCT116R and HT29R); subsequently, radiation (2 Gy) in combination with CBD treatment overcame radioresistance via the modulation of the epithelial–mesenchymal transition (EMT) phenomenon, including the increase in N-cadherin and vimentin and the reduction in E-cadherin. Thus, these results show that CBD may be a new powerful therapeutic approach for CRC radiotherapy.

## 1. Introduction

Colorectal cancer (CRC) incidence is significant globally; it was the third most common cancer worldwide in 2022, with over 1.9 million new cases reported [1]. Moreover, CRC is the second most common diagnosed cancer type and cause of cancer death in both sexes [2]. CRC is frequently treated with combination therapies, including surgery, immunotherapy, chemotherapy, and radiotherapy, depending on the cancer location and stage [3]. Radiotherapy is a potential tumor therapeutic strategy for CRC, but it often induces radioresistance [4].

Radiotherapy for CRC is a general therapy using protons beams, high-energy X-rays, and particle beams of ionizing radiation to destroy CRC cells [5]. In radiotherapy for cancer, radioresistance is associated with complex mechanisms, such as genetic and epigenetic alterations, that often promote the epithelial–mesenchymal transition (EMT) [6]. In cancer radiotherapy, radioresistance regulates EMT markers through the activation of several EMT-related pathways, including TGFβ, Wnt/β-catenin, Notch, PI3K/Akt/mTOR, ERK, Glycolysis, and VEGF [7]. Radioresistance by EMT also are modulated by EMT-related transcription factors, including Slug, Snail, and Twist [8]. Combined chemotherapy and radiotherapy (chemo-radiotherapy) is a powerful approach for overcoming radioresistance [9,10]. Radiation causes damage to both normal and cancer cells, but kills cancer cells more preferentially than normal cells [11]. Hypoxic microenvironment in cancer radiotherapy mediates radioresistance via the activation of hypoxic inducible factor-1 (HIF-1)-related pathway and EMT markers, such as snail, slug, and twist [12]. Accumulating studies have indicated that radiation in combination with EMT targeting natural compounds is a potential cancer therapeutic strategy to sensitize radioresistant cancer cells [13].

*Trichosanthes kirilowii* has long been used in traditional herbal medicine [14]. Cucurbitacin D (CBD) is a tetracyclic triterpenoid and one of the bioactive compounds extracted from *Trichosanthes kirilowii* [15]. CBD exerts several biological effects, including anti-cancer and –inflammatory activities [16]. Recent reports have indicated that CBD mediates an anti-inflammatory effect by inhibiting the levels of nitric oxide (NO), cyclooxygenase-2 (COX-2), and inducible nitric oxide synthase (iNOS) in HepG2 cells [17]. CBD also induces caspase-dependent apoptotic cell death by modulating cell cycle arrest-related markers, including E6, Cyclin D1, CDK4, p-Rb, Rb, p21, and p27 in cervical and hepatocellular cancer cells [18,19].

The endoplasmic reticulum (ER) plays several potential roles, such as protein folding by chaperone proteins, protein synthesis, and calcium storage and signaling, but the failure of ER’s function triggers misfolded or unfolded protein-mediated unfolded protein response (UPR) [20]. In ER lumen, ER stress occurs when problems with ER homeostasis arise, leading to the activation of the UPR [21]. To sense ER stress in ER, there are three UPR sensors, including PKR-like ER kinase (PERK), activating transcription factor 6 (ATF6), and inositol requiring enzyme 1α (IRE1α) [22]. In unstressed general environment, the 78 kDa glucose-regulated protein (GRP78) interacts and binds to UPR sensors, but it dissociates in ER stress response and then UPR sensors activate signaling pathway, respectively [23]. The activation of UPR sensors contributes to cell survival via the regulation of cell homeostasis, but prolonged or excessive ER stress activates apoptotic cell death [24]. Of UPR sensors, the activation of PERK typically causes autophosphorylation and the resulting phosphorylation of PERK, which then it induces the phosphorylation of eIF2α [25]. The activation of eIF2α then promotes the selective translation of cytosolic ATF4, leading to its nuclear translocation, where it binds to the CHOP promoter to activate CHOP expression [26]. Finally, CHOP activates caspase-dependent apoptotic cell death, partly through the binding to death receptor 5 (DR5) promoter [27].

High reactive oxygen species (ROS) levels are an important bridge between ER stress and apoptosis, leading to Ca^2+^ release and oxidative stress [28]. The NADPH oxidase (NOX) family, such as Nox1–5, Nox/dual oxidase 1 (DUOX1), and DUOX2, is a major source of ROS production [29]. Nox2-mediated ROS induces ER stress and apoptotic cell death through the activation of pro-apoptotic CHOP-Ca^2+^/calmodulin-dependent protein kinase II (CaMKII) signaling axis [30]. ROS release caused by Nox4 activates the PERK-eIF2α-CHOP axis, inducing apoptosis [31].

In this research, we suggested that CBD mediates anti-inflammatory and anti–cancer activity via NOX4-mediated ER stress response and apoptotic cell death in colorectal cancer cells. In addition, CBD sensitizes radioresistant colorectal cancer cells to radiation.

## 2. Results

### 2.1. CBD Treatment Mediates Anti-Inflammatory Effect in LPS-Induced Macrophage and Sepsis Mouse Models

**Figure 1:**
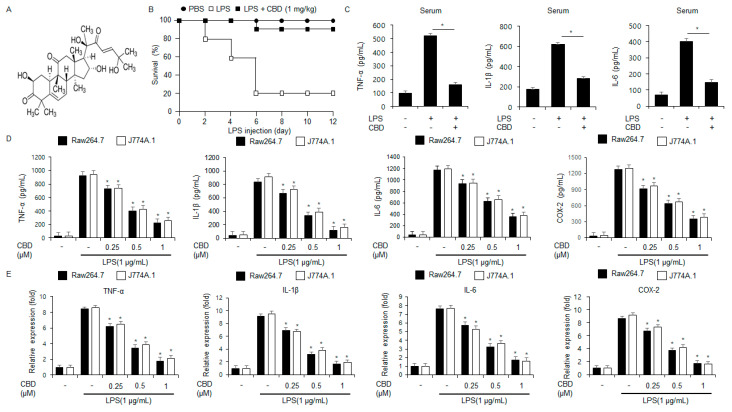
CBD modulates mRNA and protein expression of inflammatory cytokines in LPS-mediated macrophage. (**A**) The chemical structure of CBD. (**B**) To evaluate survival outcomes, C57BL/6 mice were administered LPS (20 mg/kg i.p.) or a combination of LPS and CBD (1 mg/kg i.p.). Survival rates for the LPS-only and co-treated groups (n = 10/group) were monitored daily for 12 days. (**C**) Effects of CBD on serum protein levels of IL-1β, TNF-α, and IL-6 in LPS-mediated mouse models, measured by ELISA. (**D**,**E**) ELISA and Real-time qPCR were used to quantify the mRNA and protein expression of IL-1β, IL-6, COX-2, and TNF-α in J774A.1 and Raw264.7 cells. The cells were pre-treated with LPS (1 μg/mL) and co-treated with CBD (0, 0.25, 0.5, and 1 µM) for 24 h. The relative mRNA levels were normalized using β-actin. * = *p* < 0.05. These experiments were repeated three times.

This is the chemical structure of CBD (Figure 1A). To identify the anti-inflammatory efficacy of CBD, in vivo and in vitro experiments using LPS-induced immortalized murine macrophage-like cell type and sepsis mice models were performed. Combined LPS and CBD (1 mg/kg) treatments resulted in an approximately 4- to 5-fold increase in survival rate compared to LPS alone (Figure 1B). To confirm the anti-inflammatory activity of CBD in LPS-induced sepsis mouse model, we investigated the protein expression levels of inflammatory cytokines, such as TNF-α, IL-6, and IL-1β from the sepsis mouse serum samples. In the ELISA, CBD treatment indicated the downregulation of TNF-α, IL-6, and IL-1β expression levels in the serum samples of the LPS-mediated sepsis mouse models (Figure 1C). To investigate whether CBD modulates the anti-inflammatory effect in the LPS-mediated immortalized murine macrophage-like Raw264.7 and J774.1 cell lines, ELISA and qPCR assays were carried out. After J774.1 and Raw264.7 cells were pretreated with LPS, CBD markedly decreased the levels of IL-1β, IL-6, COX-2, and TNF-α in a dose-dependent manner (Figure 1D,E). These results showed that combined CBD and LPS suppressed the increase in inflammatory cytokines, including IL-6, TNF-α, COX-2, and IL-1β, in LPS-induced immortalized murine macrophage-like J774.1 and Raw264.7 cell lines.

### 2.2. CBD Treatment Leads to Caspase-Mediated Apoptosis in Colorectal Cancer Cells

**Figure 2:**
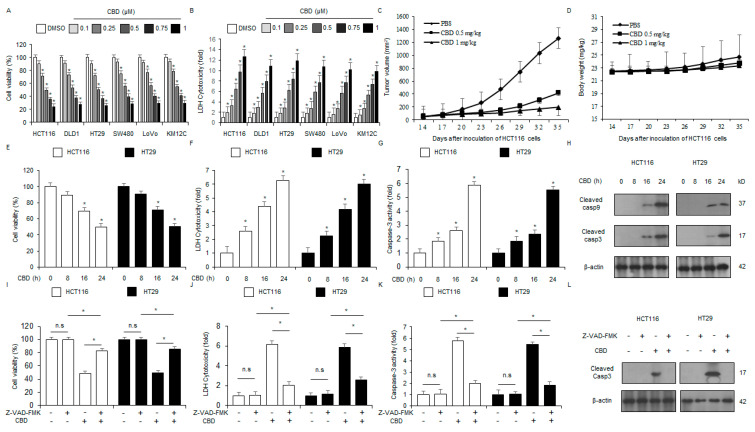
The anti-proliferative effects of CBD on colorectal cancer cells and xenograft mouse model. (**A**,**B**): Cell viability and cytotoxicity were evaluated in CBD-treated colorectal cancer cell lines (HCT116, HT29, DLD1, LoVo, and KM12C) using the WST-1 and LDH assays, respectively, after 24 h at the indicated concentrations (0, 0.1, 0.25, 0.5, 0.75, and 1 µM). (**C**,**D**): A xenograft model was established by subcutaneously implanting 1 × 10^7^ HCT116 cells into the right dorsal flank of nude mice (n = 10/group). Treatment with CBD (0.5 and 1 mg/kg) was performed through intraperitoneal administration twice per week. We monitored the body weights of the xenograft mice models twice weekly throughout the experiment. (**E**–**H**) Following treatment with 0.5 μM CBD at indicated time points (0, 8, 16, and 24 h), after which we conducted several experiments, including analyses of caspase-3 activity, LDH release, and WST-1 viability. Western blot analysis indicated time-dependent cleavage of caspase-9 and -3 in HCT116 and HT29 cells following CBD treatment; *, *p* < 0.05. The protein levels were normalized using β-actin. (**I**–**L**) HCT116 and HT29 cell lines were first pre-incubated with Z-VAD-FMK (50 μM) for 4 h and then treated with CBD (0.5 µM) for 24 h. We then conducted a series of biological experiments, such as WST-1, LDH assay, and caspase-3 activity; *, *p* < 0.05, n.s = no significance. The presence of cleaved caspase-3 was determined via Western blot assay, utilizing a cleaved caspase-3 antibody. The protein levels were normalized using β-actin.

To evaluate the anti-cancer efficacy of CBD in a panel of colorectal cancer cell lines (HCT116, DLD1, HT29, SW480, LoVo, and KM12C), we carried out dose–response experiments using cytotoxicity LDH and WST-1 assays with CBD concentrations of 0.1, 0.25, 0.5, 0.75, and 1 µM. CBD treatment was found to reduce cell viability and enhance LDH cytotoxicity across various colorectal cancer cell lines (Figure 2A,B). To further investigate the anti-tumor activity of CBD in vivo, we conducted the experiments using xenograft nude mouse model. Following the establishment of HCT116 xenografts, mice received CBD injections at doses of 0.5 mg/kg and 1 mg/kg. This finding suggested that CBD treatment resulted in lower tumor volumes compared with the control (Figure 2C). There was no significant body weight loss in any of the groups treated with CBD (Figure 2D). To assess the time-dependent anti-tumor effects of 100 µM CBD, HCT116 and HT29 cells underwent treatment for 0, 8, 16, and 24 h, followed by analysis using LDH, caspase-3 activity, and WST-1 assays. CBD treatment significantly increased caspase-3 activity and LDH cytotoxicity while simultaneously decreasing cell viability in a time-dependent manner (Figure 2E–G). Western blotting demonstrated the time-dependent induction of caspase-9 and -3 cleavage by CBD treatment (Figure 2H). To investigate the role of the caspase-dependent pathway in CBD-mediated apoptosis in HCT116 and HT29 cells, we conducted assays in the presence of the pan-caspase inhibitor Z-VAD-FMK. No changes in LDH cytotoxicity, caspase-3 activity, and cell viability were observed with Z-VAD-FMK (50 μM) treatment alone. The combination of Z-VAD-FMK (50 μM) and CBD (0.5 µM) led to a significant suppression of elevated LDH and caspase-3 activity and reduced cell viability (Figure 2I–K). Compared to treatment with CBD alone, the addition of Z-VAD-FMK diminished the levels of caspase-3 cleavage in Western blot results (Figure 2L). The results indicate that CBD treatment suppresses colorectal cancer cell growth, a process mediated by caspase-dependent apoptosis.

### 2.3. CBD Induces Apoptosis in Colorectal Cancer Cells via the Induction of ER Stress

**Figure 3:**
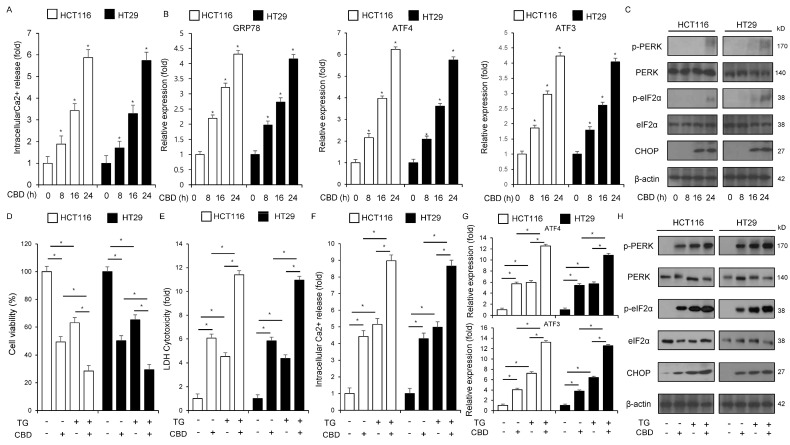
CBD triggers apoptosis through the induction of ER stress and subsequent intracellular Ca^2+^ production. (**A**) Intracellular Ca^2+^ concentrations were measured in HCT116 and HT29 cells after treatment with 0.5 µM CBD for 0, 8, 16, and 24 h; *, *p* < 0.05. (**B**) qRT-PCR was used to calculate the mRNA expression levels of ATF3, ATF4, and GRP78. The protein levels were normalized using β-actin. (**C**) HCT116 and HT29 cells were exposed to 0.5 µM CBD for 0, 8, 16, and 24 h to analyze the ER stress signaling pathway. Western blotting was used to determine the protein expression of CHOP, PERK, eIF2α, and the phosphorylation of PERK and eIF2α. The protein levels were normalized using β-actin. (**D**–**F**) Following treatment with 3 μM thapsigargin (TG) and 0.5 µM CBD for 24 h, HCT116 and HT29 cells were used for subsequent experiments. We conducted several biological assays, such as the LDH, intracellular Ca^2+^, and WST-1 assays; *, *p* < 0.05. (**G**,**H**) qRT-PCR was used to confirm the mRNA levels of ATF3 and ATF4, while Western blot analysis was conducted to examine the protein expression levels of p-eIF2α, eIF2α, p-PERK, PERK, and CHOP in HCT116 and HT29 cells treated with 0.5 µM CBD or 3 μM TG for 24 h. The protein levels were normalized using β-actin.

High concentrations and excessive influx of Ca^2+^ lead to apoptosis, despite Ca^2+^’s normal role in modulating various cellular processes such as homeostasis, proliferation, cancer immunity, migration, cell death, and survival [32,33]. Upon Ca^2+^ release within the ER lumen, an ER stress response is induced, subsequently mediating apoptosis [34]. To investigate whether CBD mediates time-dependent intracellular Ca^2+^ release in HCT116 and HT29 cell lines, we conducted experiments using an intracellular Ca^2+^ assay. The finding demonstrated that CBD triggers a time-dependent release of intracellular Ca^2+^. As shown in Figure 3A, CBD treatment induced time-dependent changes in HCT116 and HT29 cells, as confirmed by qRT-PCR and Western blot. The time-dependent mRNA levels of CHOP, ATF3, and ATF4, and the protein levels of p-PERK, p-eIF2α, and CHOP were enhanced by CBD treatment (Figure 3B,C). To determine if the ER stress response contributes to CBD-mediated apoptosis, HCT116 and HT29 cell lines were co-treated with the ER stress inducer thapsigargin (TG) and CBD. Experiments were subsequently conducted using assays for intracellular Ca^2+^ activity, WST-1, and cytotoxicity LDH. The combination of TG and CBD resulted in a synergistic decrease in cell viability (Figure 3D–F). Conversely, this combined treatment significantly increased intracellular Ca^2+^ production and LDH cytotoxicity (Figure 3D–F). In qRT-PCR and Western blot assays, combined treatment with TG and CBD synergistically enhanced the levels of ATF4, CHOP, and ATF3, as well as the phosphorylation of PERK and eIF2α (Figure 3G,H).

### 2.4. Targeting PERK or CHOP Suppresses CBD-Mediated Apoptosis in Colorectal Cancer Cells

**Figure 4:**
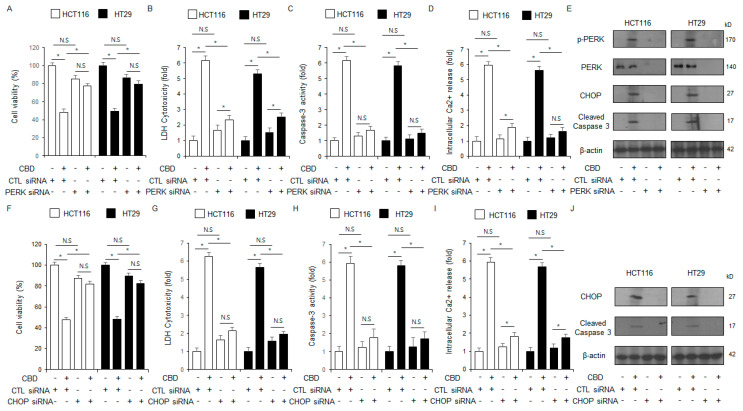
Silencing PERK expression reduces CBD-mediated apoptosis in colorectal cancer cells. (**A**–**E**) Following transfection with PERK siRNAs, HCT116 and HT29 cells were treated with CBD (0.5 µM) for 24 h. We assessed caspase-3 activity, intracellular Ca^2+^ levels, and performed both WST-1 and LDH assays to evaluate cell death and viability; *, *p* < 0.05, N.S = no significance. Protein expression levels of cleaved caspase-3, CHOP, and PERK, and phosphorylation levels of PERK were determined by Western blot analysis in HCT116 and HT29 cells after 24 h of 0.5 µM CBD treatment. The protein levels were normalized using β-actin. (**F**–**J**) Following transfection with CHOP siRNAs, HCT116 and HT29 cells were treated with CBD (0.5 µM) for 24 h. We assessed caspase-3 activity, intracellular Ca^2+^ levels, and performed both WST-1 and LDH assays to evaluate cell death and viability; *, *p* < 0.05, N.S = no significance. Protein expression levels of cleaved caspase-3 and CHOP were determined by Western blot analysis in HCT116 and HT29 cells after 24 h of 0.5 µM CBD treatment. The protein levels were normalized using β-actin.

To identify whether the loss of PERK regulates CBD-mediated apoptotic cell death in HCT116 and HT29 cell lines, we transfected these cells with PERK-specific siRNAs and then treated them with CBD. We further investigated the effects by measuring intracellular Ca^2+^ activity, cytotoxicity via LDH and WST-1 assays, caspase-3 activity, and performing a Western blot analysis. Our results indicate that PERK deficiency prevents the enhancement of caspase-3 activity, intracellular Ca^2+^ levels, and LDH release, while simultaneously improving cell viability (Figure 4A–D). Western blot results showed that PERK knockdown decreased the phosphorylation of PERK, the cleavage of caspase-3, and the expression of CHOP in CBD-induced HCT116 and HT29 cells relative to the control (Figure 4E). To determine whether CHOP, a downstream effector of the PERK pathway, regulates CBD-mediated apoptosis, HCT116 and HT29 cell lines were transfected with CHOP-specific siRNAs prior to CBD treatment. We investigated the effects using intracellular Ca^2+^ activity, LDH cytotoxicity, caspase-3 activity, WST-1 assays, and Western blot analysis. CHOP knockdown attenuated the elevation in caspase-3 activity, intracellular Ca^2+^ levels, and LDH release, and also mitigated the reduction in cell viability (Figure 4F–I). Western blotting revealed that the reduction in cleaved caspase-3, and CHOP levels in CBD-induced HCT116 and HT29 cells was mediated by CHOP knockdown (Figure 4J). The ER stress signaling cascade was demonstrated to induce apoptosis in colorectal cancer cells treated with CBD.

### 2.5. Targeting NOX4 Reverses the Apoptotic Process Normally Initiated by CBD Treatment in Colorectal Cancer Cells

**Figure 5:**
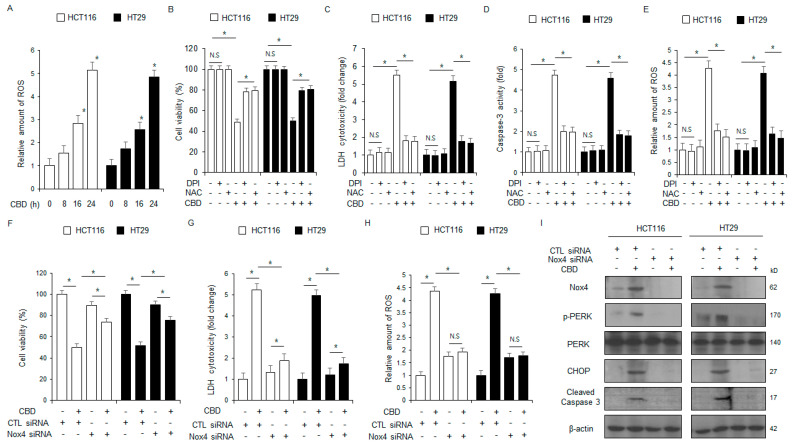
Silencing of NOX4 prevents ROS-induced ER stress and apoptosis in CBD-treated colorectal cancer cells. (**A**) Intracellular ROS generation was measured using the DCFDA dye in HCT116 and HT29 cells after treatment with 0.5 μΜ CBD for the indicated durations; *, *p* < 0.05. (**B**–**E**) Following treatment with 0.5 μΜ CBD, 1 μM DPI, and 100 μM NAC for 24 h, HCT116 and HT29 cells were subjected to various assays, including those for intracellular ROS release, caspase-3 activity, WST-1, and LDH cytotoxicity; *, *p* < 0.05, N.S = no significance. (**F**–**I**) Following transfection of NOX4 siRNAs into HCT116 and HT29 cell lines, cells were treated with CBD (0.5 µM) for 24 h. A variety of assays were performed, including those for intracellular ROS release, LDH cytotoxicity, and WST-1; *, *p* < 0.05. Protein expression levels of PERK, p-PERK, NOX4, CHOP, and cleaved caspase-3 were identified by Western blot analysis in HCT116 and HT29 cells after 24 h of 0.5 µM CBD treatment. The protein levels were normalized using β-actin.

To assess whether CBD treatment affects the release of intracellular ROS in colorectal cancer cells, we conducted experiments using intracellular ROS assays. Intracellular ROS production increases over time following CBD treatment (Figure 5A). We investigated whether the effects of CBD treatment are mediated by intracellular ROS release; to do this, we treated HCT116 and HT29 cells with ROS inhibitors (DPI and NAC) after CBD exposure and conducted various assays (LDH, intracellular ROS, caspase-3 activity, and WST-1). The combination of CBD with NAC or DPI inhibited the increase in LDH cytotoxicity, intracellular ROS release, caspase-3 activity, and prevented the decrease in cell viability more effectively than CBD treatment alone (Figure 5B–E). We performed transfection experiments using NOX4-specific siRNAs in HCT116 and HT29 cell lines, followed by CBD treatment, to determine if ROS production is linked to NOX4 expression. We then assessed the cells using intracellular ROS, LDH cytotoxicity, WST-1, and Western blot analysis. The data indicates that NOX4 knockdown is crucial for inhibiting the increase in intracellular ROS and LDH cytotoxicity, and maintaining cell viability (Figure 5F–H). The results from Western blot analysis demonstrated that NOX4 knockdown suppressed the elevation of cleaved caspase-3, p-PERK, CHOP, and NOX4 levels in CBD-treated HCT116 and HT29 cells relative to controls (Figure 5I). Our findings suggest that Nox4 is involved in CBD-mediated intracellular ROS release and plays a role in regulating CBD-induced apoptosis in colorectal cancer cells.

### 2.6. Both Radiation and CBD Treatment Reduce Radioresistance in Radioresistant Colorectal Cancer Cells to Radiation by Modulating the EMT Phenomenon

**Figure 6:**
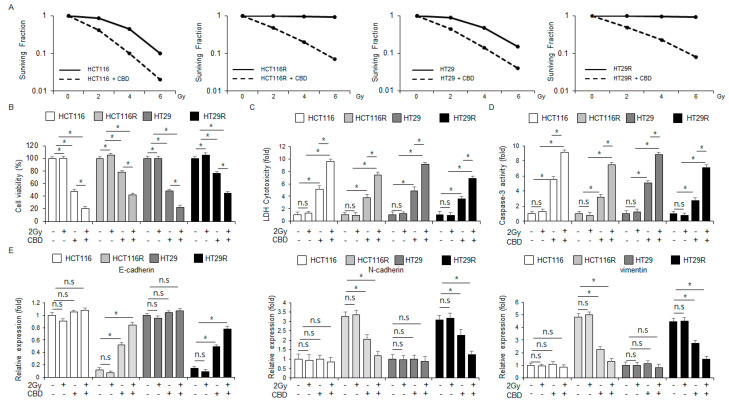
Combining CBD with radiation enhances radiosensitivity in radio-resistant colorectal cancer cell lines. (**A**) To assess clonogenic survival, HCT116, HT29, HCT116R, and HT29R cells were irradiated on the indicated conditions (0, 2, 4, and 6 Gy) in combination with CBD treatment. The resulting survival fractions were then calculated; *, *p* < 0.05. (**B**–**D**) We treated HCT116, HT29, HCT116R, and HT29R cells with a combination of 0.5 µM CBD (24 h) and 2 Gy radiation (24 h). We then performed a variety of assays, such as caspase-3 activity, WST-1, and LDH cytotoxicity. (**E**) We treated HCT116, HT29, HCT116R, and HT29R cells with a combination of 0.5 µM CBD and 2 Gy radiation for 24 h and then performed real-time qPCR to examine the mRNA levels of N-cadherin, E-cadherin, and vimentin.; *, *p* < 0.05, n.s = no significance. The mRNA levels were normalized using β-actin.

Despite its effectiveness in treating colorectal cancer, radiotherapy often leads to the development of radioresistance [35]. To assess the ability of CBD to overcome radioresistance in HCT116R and HT29R cells, we conducted experiments using colony formation assays. The surviving fractions for HCT116, HCT116R, HT29, and HT29R cells were decreased more effectively by CBD treatment at 2, 4, and 6 Gy compared to the controls (Figure 6A). In both HCT116 and HT29 cell lines, CBD treatment alone reduced cell viability while enhancing LDH cytotoxicity and caspase-3 activity (Figure 6B–D). When radiation (2 Gy) was combined with CBD, these effects were enhanced even further (Figure 6B–D). Notably, radiation (2 Gy) administered alone had no significant effect on caspase-3 activity, LDH cytotoxicity, or cell viability (Figure 6B–D). While CBD treatment alone was effective in reducing cell viability and increasing LDH cytotoxicity and caspase-3 activity in HCT116R and HT29R cells, the combination with 2 Gy radiation produced an even more pronounced effect (Figure 6B–D). Radiation (2 Gy) by itself did not alter cell viability, LDH cytotoxicity, or caspase-3 activity (Figure 6B–D). We used real-time RT-PCR to examine whether the combined treatment of CBD and 2 Gy radiation regulates the EMT phenomenon in radio-resistant HCT116R and HT29R colorectal cancer cells. The qRT-PCR results demonstrated that the mRNA levels of E-cadherin were reduced and the mRNA levels of N-cadherin and vimentin were increased in HCT116R and HT29R cells, indicating the change in the EMT phenotype in radio-resistant colorectal cancer cells. However, the treatment with either CBD alone or the combination of 2 Gy radiation and CBD enhanced the mRNA levels of E-cadherin and reduced the levels of vimentin and N-cadherin in HCT116R and HT29R cells (Figure 6E). No significant differences were detected in the mRNA levels of N-cadherin, vimentin, and E-cadherin in the HCT116 and HT29 cell lines (Figure 6E). Our results suggest that a combination therapy of radiation and CBD may represent a novel strategy to overcome radioresistance in HCT116R and HT29R cells by altering EMT markers.

## 3. Discussion

According to recent research, natural compounds such as flavonoids, alkaloids, terpenoids, phenols, and exhibit potent anti-cancer activity in cancer therapy [36,37]. A common mechanism for natural compound to induce cancer cell death is through the generation of high intracellular levels of Ca^2+^ and ROS, which mediate apoptosis via oxidative stress, suggesting a promising role for redox-modulating agents in cancer therapy [38,39]. Here, we demonstrate that the natural bioactive compound CBD is a promising anti-tumor agent that sensitizes radioresistant colorectal cancer cells to radiotherapy. The results indicate that CBD induces an anti-cancer effect by activating the apoptosis signaling pathway in colorectal cancer cells under both in vitro and in vivo conditions. Induction of the apoptosis signaling pathway by natural products is a potential anti-cancer mechanism to inhibit tumor growth and proliferation [40]. The combination of CBD and Z-VAD-FMK inhibited caspase-dependent apoptosis in colorectal cancer cells, suggesting that CBD acts via the apoptosis signaling pathway. Activation of ER stress sensors (PERK, IRE1α, and ATF6) by excessive ER stress initiates downstream signaling cascades that lead to apoptosis [41]. Upon UPR induction, the GRP78/Bip dissociates from ER stress sensors, after which the autophosphorylation of PERK leads to the phosphorylation of eIF2α [42]. Through the phosphorylation of eIF2α, the levels of ATF4 and ATF3 increase in the cytosol. This results in the translocation of ATF4 to the nucleus, where it binds the CHOP promoter and upregulates CHOP expression [43]. The release of intracellular Ca^2+^ and ROS by CBD activates the ER stress signaling cascade. This activation, specifically through the PERK-CHOP signaling pathway, ultimately leads to apoptosis. These findings suggest that CBD induces apoptosis through the PERK-CHOP axis, as evidenced by suppressed apoptosis following PERK or CHOP knockdown and synergistic apoptosis with combined TG treatment in colorectal cancer cells. NOX4 acts as a powerful modulator of CBD-induced ROS production, and its subsequent activation induces ER stress-induced apoptosis via intracellular ROS and Ca^2+^ release in CBD-treated colorectal cancer cell lines. Through inhibitory experiments, it was observed that NOX4 knockdown and DPI orNAC treatment block various cellular responses, including specifically intracellular ROS release, caspase-3 activity, LDH cytotoxicity, and the PERK-CHOP cascade, in colorectal cancer cells. Despite the purpose of radiotherapy being the elimination of tumor cells, the exposure to radiation often leads to these cells acquiring resistance to further treatment [44]. According to recent reports, natural radiosensitizers, including taxanes, vinblastine, curcumin, and quercetin, serve as effective adjuvants in cancer patient radiotherapy and cause fewer side effects [45,46]. The EMT process is linked to radioresistance and chemoresistance under hypoxia conditions [47]. Novel anti-cancer agents designed to overcome radioresistance may be crucial in reducing the failure of radiotherapy. Combining CBD with radiation sensitizes the radioresistant colorectal cancer cell lines HCT116R and HT29R through the modification of EMT markers, including E-cadherin, vimentin, and N-cadherin.

Numerous natural compounds exhibit potent anti-cancer effects across a range of cancer types [48]. Ginsenoside compound K, a natural product, induces an apoptosis cascade in human colon cancer cells by upregulating DR5, cleaved caspase-9 and -3 and downregulating BCL2 expression [49]. The PERK-CHOP axis-mediated ER stress and apoptosis induced by celastrol, a bioactive compound from *Tripterygium wilfordii*, induces ER stress-mediated apoptosis through the PERK-CHOP signaling cascade in gastric cancer cells; this effect in SGC-7901 and BGC-823 gastric cancer cells are inhibited when combined with the ROS scavenger NAC treatment [50]. The inhibition of EMT markers like E-cadherin, vimentin, and N-cadherin by curcumin contributes to its anti-cancer effects and the sensitization of radioresistance in Panc-1 pancreatic cancer cells [51]. The results indicate that CBD mediates apoptosis via the PERK-CHOP axis in colorectal cancer cell lines. When combined with radiation, CBD sensitizes radioresistance in HCT116R and HT29R cells through the regulation of EMT markers, including N-cadherin, E-cadherin, and vimentin. A recent study suggested that CBD treatment leads to caspase-3 dependent apoptosis in H1299 human NSCLC cells through the inhibition of NF-κB, p-ERK, STAT3, p-JNK, and p-AKT [52].

In various cell types, the upregulation of ATF4 and CHOP due to ROS release and mitochondrial dysfunction leads to ER stress-mediated apoptosis [53]. As a key source of ROS generation NADPH oxidases (NOXs) play a potential role in regulating cell metabolism within the context of cancer [54,55]. NOXs are a family of seven membrane-bound enzymes that produce superoxide radicals and are known to be involved in different pathophysiological disorders, such as neurodegeneration, inflammation, cancer, and cardiovascular disease [56]. Both the modulation of mitochondrial NOX4 activity by ATP and its role as a key determinant of cell survival and cell death are significant findings [57]. Curcumin mediates NOX4-induced mitochondrial ROS and then it also induces caspase-dependent apoptotic cell death through the reduction in c-FLIP and Mcl-1 expression in head and neck squamous cell carcinoma, human breast cancer, and human glioma cells [58]. CBD triggers caspase-dependent apoptosis via the PERK-CHOP cascade, with NOX4-derived ROS release serving as the critical mediator. In CBD-treated colorectal cancer cells, cell viability was maintained, and the levels of intracellular ROS, cytotoxicity LDH, and caspase-3 activity were suppressed when NOX4 was knocked down or when DPI or NAC was administered.

Radiotherapy employs high-energy beams to destroy or damage cancer cells [59]. To reduce radiotherapy failure caused by radioresistance in colorectal cancer, combination therapy is suggested as a potentially significant treatment option [60]. When exposed to radiation, cancer cells in a hypoxic environment often acquire radioresistance by modifying the epithelial–mesenchymal transition (EMT) phenomenon [61]. During the EMT process, the epithelial cell marker E-cadherin showed reduced expression, while the mesenchymal markers N-cadherin and vimentin were upregulated [62]. Our results indicate that CBD and radiation in combination reverse radioresistance in HCT116R and HT29R colorectal cancer cells by altering EMT characteristics, including elevated vimentin and N-cadherin expression and reduced E-cadherin expression.

## 4. Materials and Methods

### 4.1. Reagents

Cucurbitacin D (CBD; PHL80254), Thapsigargin (TG; Millipore, Bedford, MA, USA; T9033), Z-VAD-FMK, Diphenyleneiodonium chloride (DPI; D2926), Lipopolysaccharide (LPS; L4391), and N-acetylcysteine (NAC; A9165) were purchased from Sigma-Aldrich (Sigma-Aldrich, St. Louis, MO, USA).

### 4.2. Cell Culture

The J774.1 and Raw264.7, immortalized murine macrophage-like cell type, were purchased from the American Type Culture Collection (Rockville, MD, USA) and cultured and maintained in Dulbecco’s Modified Eagle Medium (DMEM; Welgene, Gyeongsan, Republic of Korea) with 10% inactivated Fetal Bovine Serum (FBS; Gibco, Grand Island, NY, USA) and 1% penicillin-streptomycin (PS; Gibco, Grand Island, NY, USA). HCT116, DLD1, HT29, SW480, LoVo, and KM12C cells, human colorectal cancer cell lines, were purchased from the Korean Cell Line Bank (Cancer Research Center, Seoul National University, Seoul, Republic of Korea). Human colorectal cancer cells were cultured in Rosewell Park Memorial Institute (RPMI) 1640 and DMEM mediums (Welgene, Gyeongsan, Republic of Korea) containing 10% inactivated FBS (FBS; Gibco, Grand Island, NY, USA), 1% penicillin-streptomycin (PS; Gibco, Grand Island, NY, USA) at 37 °C with 5% CO_2_.

### 4.3. Cell Viability Assay

Colorectal cancer cells (1 × 10^4^ cells/well) were seeded into 96-well cell culture plate. Cell viability was performed using a cell viability reagent WST-1 solution (Sigma-Aldrich, St. Louis, MO, USA). The absorbance was analyzed at 450 nm using a microplate reader (Molecular Devices, San Jose, CA, USA). This experiment was performed following the manufacturer’s instructions.

### 4.4. LDH Cytotoxicity Assay

Colorectal cancer cells (1 × 10^4^ cells/well) were seeded and maintained into 96-well cell culture plate. Lactate dehydrogenase (LDH) cytotoxicity was carried out using a LDH cytotoxicity assay (Dojindo Laboratories, Kumamoto, Japan). The absorbance was analyzed at 490 nm using a fluorescence microplate reader FilterMax F5 (Molecular Devices, San Jose, CA, USA). This experiment was performed following the manufacturer’s instructions.

### 4.5. Caspase-3 Activity Assays

Colorectal cancer cells (1 × 10^4^ cells/well) were seeded and maintained into 96-well cell culture plate. Caspase-3 activity was carried out using a Caspase-3 activity assay (Cell Signaling Technology, Boston, MA, USA). The absorbance was analyzed at Ex/Em = 380/460 nm using a fluorescence microplate reader FilterMax F5 (Molecular Devices, San Jose, CA, USA). This experiment was performed following the manufacturer’s instructions.

### 4.6. Intracellular Ca^2+^ Assays

Colorectal cancer cells (1 × 10^4^ cells/well) were seeded and maintained into 96-well cell culture plate. Intracellular Ca^2+^ activity was carried out using an intracellular Ca^2+^ assay (Sigma-Aldrich, St. Louis, MO, USA). The absorbance was analyzed at 575 nm using a fluorescence microplate reader FilterMax F5 (Molecular Devices, San Jose, CA, USA). This experiment was carried out following the manufacturer’s instructions.

### 4.7. Intracellular ROS Assays

Colorectal cancer cells (1 × 10^4^ cells/well) were seeded and maintained into 96-well cell culture plate. Intracellular ROS production was carried out using an intracellular ROS assay (Sigma-Aldrich, St. Louis, MO, USA). The absorbance was analyzed at Ex/Em = 490/520 nm using a fluorescence microplate reader FilterMax F5 (Molecular Devices, San Jose, CA, USA). This experiment was carried out following the manufacturer’s instructions.

### 4.8. Establishment of Radioresistant HCT116 and HT29 Cell Lines

HCT116 and HT29 cell lines were plated and maintained into 60 mm cell culture dishes. For radiation exposure, the cells were irradiated at the indicated condition (4 Gy) daily for 12 weeks after 24 h. To establish radioresistant HCT116 and HT29 cells, this experiment was repeated and then radioresistant cells (HCT116R and HT29R) were established.

### 4.9. Irradiation

HCT116, HCT116R, HT29, and HT29R cell lines were plated and maintained into 60 mm dishes and incubated at 37 °C CO_2_ for 24 h. And it was irradiated from ^137^Cs source irradiation (Atomic Energy of Canada, Ltd., Mississauga, ON, Canada). Established HCT116R and HT29R cells were irradiated to a 4 Gy dose for 90 days and were cultured and maintained in a growth medium containing 10% FBS.

### 4.10. Colony Formation Assay

HCT116, HCT116R, HT29, and HT29R cell lines were plated and maintained into 60 mm dishes with growth medium. To make colony formation, cells were maintained for 2 weeks and then the colonies were stained with 0.5% crystal violet (Sigma-Aldrich, St. Louis, MO, USA). This experiment was carried out following the manufacturer’s instructions.

### 4.11. Transfection

Small interfering RNAs (siRNAs) were purchased from PERK (Bioneer, Daejeon, Republic of Korea), CHOP (Bioneer, Daejeon, Republic of Korea) and NOX4 (Bioneer, Daejeon, Republic of Korea). HCT116 and HT29 cell lines were seeded and maintained into a 6-well plate and cells were transfected with siRNAs (30 nmol/mL) using Lipofectamine 2000 reagent (Invitrogen, Waltham, MA, USA) following the manufacturer’s instructions. The CTL siRNA used was a scrambled siRNA (Santa Cruz, Dallas, TX, USA).

### 4.12. RNA and Protein Extraction

Cells were seeded and maintained into a 100 mm cell culture dish with growth medium. Total RNA from J774.1, Raw264.7, HCT116, HCT116R, HT29, and HT29R cell lines were extracted using Trizol reagent (Invitrogen, Waltham, MA, USA) following the manufacturer’s protocol. Protein of cell lysates collected by RIPA lysis buffer (Sigma-Aldrich, St. Louis, MO, USA).

### 4.13. qRT PCR and Western Blot Analyses

For Real-time qRT PCR analysis, triplicate reactions were performed. The primer sequences were as follows; IL-6 [5′-CTGATGCTGGTGACAACCAC-3′ (sense) and 5′-TCCACGATTTCCCAGAGAAC-3′ (antisense)], IL-1β [5′-GAGTGTGGATCCCAAGCAAT-3′ (sense) and 5′-CTTGTGCTCTGCTTGTGAGG-3′ (antisense)], TNF-α [5′-ACGGCATGGATCTCAAAGAC-3′ (sense) and 5′-TGAGATAGCAAATCGGCTGAC-3′ (antisense)], COX-2 [5′-CCACTTCAAGGGAGTCTGGA-3′ (sense) and 5′-AGTCATCTGCTACGGGAGGA-3′ (antisense)], GRP78 [5′-TCAGCCCACCGTAACAAT-3′ (sense) and 5′-CAAACTTCTCGGCGTCAT-3′ (antisense)], ATF4 [(5′-AAGCCTAGGTCTCTTAGATG-3′ (sense) and 5′-TTCCAGGTCATCTATACCCA-3′ (antisense)], ATF3 [(5′-CGCTGGAATCAGTCACTGTCAG-3′ (sense) and 5′-CTTGTTTCGGCACTTTGCAGCTG-3′ (antisense)], E-cadherin [5′-GAACGCATTGCCACATACAC-3′ (sense) and 5′-GAATTCGGGCTTGTTGTCAT-3′ (antisense)], N-cadherin [5′-GGCATACACCATGCCATCTT-3′ (sense) and 5′-GTGCATGAAGGACAGCCTCT-3′ (antisense)], and vimentin [5′-GAGAACTTTGCCGTTGAAGC-3′ (sense) and 5′-GCTTCCTGTAGGTGGCAATC-3′ (antisense)] on a Roche LightCycler 96 System (Roche, Basel, Switzerland). RNA quantities were normalized with β-actin primer [5′-AAGGCCAACCGCGAGAAGAT-3′ (sense) and 5′-TGATGACCTGGCCGTCAGG-3′ (antisense)], and the relative fold change for gene expression analyses was calculated using the 2^−ΔΔCt^ method. The PVDF membranes (Millipore, Burlington, MA, USA) were blocked with 5% skim milk and then incubated with primary antibodies. The primary antibodies used included p-PERK(Thr980) (Cell Signaling, Danvers, MA, USA), PERK (Cell Signaling, Danvers, MA, USA), p-eIF2ɑ (Ser51) (Cell Signaling, Danvers, MA, USA), eIF2ɑ (Santa Cruz, Dallas, TX, USA), β-actin (Santa Cruz, Dallas, TX, USA), cleaved caspase-9 (Cell Signaling, Danvers, MA, USA), CHOP (Cell Signaling, Danvers, MA, USA), NOX4 (Proteintech, Rosemont, IL, USA), and cleaved caspase-3 (Cell Signaling, Danvers, MA, USA). And then, these were incubated with HRP-conjugated secondary antibodies, including m-IgGK BP-HRP-linked antibody (Santa Cruz, Dallas, TX, USA) and anti-mouse anti rabbit IgG HRP-linked antibody (Santa Cruz, Dallas, TX, USA). The membranes were visualized by a ECL Western Blotting Substrate (Pierce, Appleton, WI, USA).

### 4.14. Animal Experiments

Five-week-old, female, athymic BALB/c nude mice (*nu*/*nu*) were purchased from OrientBio, Inc. (Daejeon, Republic of Korea), and housed in a pathogen-free room for 1 week with NIH-7 open formula before use. The mice were divided randomly into three groups. All animal studies were performed according to the guidelines of National Institutes of Health and Kyung-Hee University Animal Care and Use Committee. For the xenograft mice experiment, 1 × 10^7^ HCT116 cells were mixed in PBS, and injected subcutaneously (sc) into right dorsal flank of nude mice. When tumor volumes reach approximately 200 mm^3^, mice were grouped randomly (n = 10 per group). And then CBD (0.5 or 1 mg/kg) was administered intraperitoneally (ip) twice weekly. Tumor volume was calculated using the following formula: (*L* × *W*^2^)/2 (mm^3^). To confirm CBD-mediated anti-inflammatory effect, mice were randomly divided into 3 groups (PBS, LPS, and LPS + CBD) and 20 mg/kg LPS induces the inflammation by intraperitoneal injection. CBD (dissolved in PBS, 1 mg/kg) was administered intraperitoneally to the treatment group immediately following LPS injection. After the injection, mice were monitored for 12 days, and blood and tissues samples were collected. In addition, the survival rate was observed and recorded for 2~12 days after the LPS challenge.

### 4.15. Cytokine Analysis

Raw264.7 and J774.1 cells (1 × 10^4^ cells/well) were seeded and cultured into a 96-well plate. To measure cytokines, the cells were treated with LPS (1 μg/mL) in the absence or presence of CBD (0, 0.5, and 1 μM; 24 h) and then an enzyme-linked immunosorbent assay (ELISA) was carried out. The expression levels of cytokines, including TNF-α, IL-6, and IL-1β, were analyzed using ELISA kits IL-1β (DY-401; R&D Systems, Minneapolis, MN, USA), IL-6 (DY-406; R&D Systems, Minneapolis, MN, USA), and TNF-α (DY-410; R&D Systems, Minneapolis, MN, USA). This experiment was performed following the manufacturer’s instructions.

### 4.16. Statistical Analysis

Experimental data were identified in at least three independent experiments, and all statistical analysis were carried out using Student’s *t*-test. *p* value < 0.05 was considered statistically significant.

## 5. Conclusions

In summary, we confirmed the anti-inflammatory and anti-cancer effects of CBD both in vitro and in vivo. CBD promotes apoptosis in colorectal cancer cell lines through a mechanism involving the PERK-CHOP signaling pathway, intracellular Ca^2+^ and ROS production, and the upregulation of NOX4. Furthermore, we show that in radiation-resistant HCT116R and HT29R cell lines, a combined approach using CBD and radiation mitigates radioresistance by modifying the EMT phenomenon.

## Data Availability

The original contributions presented in this study are included in the article. Further inquiries can be directed to the corresponding author.
